# Physiology

**Published:** 1838-01

**Authors:** 


### PART FOURTH.

### Selections from the British Journals.

(FOR THE QUARTER ENDING NOVEMBER 30, 1837.)

---

### PHYSIOLOGY.

*On the Acoustic Principles of the Stethoscope.* By CHARLES COWAN, M.D.

CONSIDERABLE difference of opinion has always prevailed respecting the cause and mode of transmission of the pectoral sounds in auscultation, and, consequently, respecting the principles on which the stethoscope should be constructed. By common consent, however, the perforated wooden cylinder of Laennec has been received as the essential type of all, however varying in external form; and it has been generally considered that the bore, open at both ends, was very important in transmitting the respiratory sounds by means of the vibrations of the air contained in it. In the present paper, however, Dr. Cowan expresses doubts both of the principle and of the alleged fact. We have only room to notice here some of the results of numerous experiments performed by him, and the conclusion to which he has arrived respecting the principle on which the stethoscope acts, and its best form. We invoke the aid of Dr. Charles Williams, to extricate us from the difficulties into which Dr. Cowan has thrown us.

"1st. If one or both ends of the stethoscope are accurately closed with cork, the respiratory murmur is only *very slightly* weakened, and the instrument would still suffice for every practical purpose.

2d. If the interval between the corks be filled with water, the sounds appeared rather louder and sharper than when the instrument was unclosed.

3d. If the cone be filled with soft cotton, and the ear end plugged with the same material, the difference in the respiratory murmur was barely appreciable.

4th. The tube was successively obstructed with dough, wax, paper, at one or both extremities; the only appreciable result was a certain diminution of the sounds, but never to such an extent as to make them not easily heard, or to neutralize the indications of the instrument. . . .

6th. When the cavity was obstructed by a silk handkerchief, it produced very little, if any, difference in the sounds: filling the tube with water considerably diminished its conducting power: leaving the extremity of the tube next to the chest open seemed rather to diminish than increase the sounds.

In all cases, accurate contact of the conducting medium with the chest and ear was indispensable. The vocal vibrations were evidently louder and more defined through the stethoscope closed at both ends and filled with water, than when open."

. . . .

"From all these facts and considerations, we feel justified in concluding that the stethoscope acts almost entirely as a solid conductor, under all circumstances, in reference to the sounds within the chest; and that the column of air which it encloses exerts little, if any, influence upon its efficiency as a conducting medium;—that the superiority of a perforated cylinder over a close one simply depends upon the former being more susceptible, by its form and lightness, of transmitting vibrations too feeble to affect a more solid instrument; but that the benefit obtained has been greatly overrated, and the explanation hitherto given erroneous.

We are inclined to believe, that the best form for a stethoscope would b a simple cylinder of light wood, about an inch and a half in diameter, and a tenth of an inch in thickness, carefully closed at both ends by a layer of the same wood, and of the same thickness. The exact length is of secondary importance. This would combine lightness and uniformity of material, a vibrating surface in contact with similar media on both sides, and great simplicity of structure."

*Med. Gazette. October* 28, 1837.

---

*On the Reaction of the Saliva upon Red and Blue Litmus and Turmeric Paper.* By T. Laycock, Esq., House-Surgeon of the York Hospital.

This is an interesting addition to the physiological subject to which it refers, and is precisely such a communication as the house-surgeons of hospitals are so well qualified to give, but which, we are sorry to say, they so rarely do give. Our readers may remember that, some time since, M. Donné made experiments on this subject, which were afterwards repeated and extended by Dr. Robert Thomson (see *British and Foreign Review,* Vol. III. p. 541.) The former gentleman stated that he never found the saliva acid when the digestion was good, and stated that acidity of the saliva is a diagnostic symptom of gastritis. Dr. Thomson found the acid reaction in all cases of inflammation of the mucous and serous membranes. With the view of testing these opinions, Mr. Laycock instituted numerous experiments, and in the present paper gives, in tables, the results of no less than 567 observations.

Mr. L.'s observations were made indiscriminately upon forty-eight in-patients of this hospital: of this number there were not three cases alike: it comprised diseased joints in every stage, ulcers, accidents, and the various other diseases usually found in a general hospital. Thirty-six, or three-fourths of the whole number, asserted that they had a good appetite and digestion. This number included the most varied cases: ulcers on the legs, with and without impaired health; diseased hip-joint with sinuses, emaciation and diarrhœa; senile gangrene; schirrous uterus, &c. Eight had appetite and digestion more or less impaired; of these, two were under the influence of mercury: four had loss of appetite, or frequent vomiting. The diet of these individuals was various: a few were on fever and full diet, but the majority on house diet, consisting of beef or mutton, and baked flour or rice puddings, on alternate days, for dinner; and tea, milk, or broth, for breakfast and supper.

The following are the principal deductions supplied by Mr. L.'s tables: they are at variance with those of M. Donné.

1. The saliva may be acid without any apparent disease of the stomach, and when the individual is in good health.

2. It is alkaline during different degrees of gastric derangement, as indicated by the tongue.

3. It may be alkaline, acid, and neutral, when the gastric phenomena are the same; and, consequently, acidity of the saliva is not a diagnostic mark of gastritic derangement.

4. In general it is alkaline in the morning, and acid in the evening.

*Med. Gazette. October* 7, 1837.

---

*Observations on the Pulses of Infants.* By J. Gorham, Esq., M.R.C.S.L.

Some short time since, Dr. William Guy read a very valuable paper "on the Effect of Posture on the Pulse," before the members of the Physical Society of Guy's Hospital, of which a very brief notice appeared in the Med. Gazette of October 28th. In this paper, which contained the results of numerous original experiments made by himself, Dr. Guy showed that the increased frequency of pulse in different positions of the body was in the ratio of the muscular action required in each case, and depended on this. We are sorry that the non-publication of Dr. Guy's paper prevents us from giving even an outline of its contents; but it has already been productive of much good, by giving occasion to the present very valuable communication by Mr. Gorham. It consists of a series of tables of the pulses of infants at different ages, and contains the results of 150 different observations, carefully made (the author informs us,) under varied circumstances and conditions of the infantile state. We can only find room for the principal conclusions deduced by Mr. Gorham from the different tables. These, however, exhibit very distinctly the general results, and show the important nature of this communication.

I. *From Birth to the age of Twenty-four Hours.* (16 Observations.)

1. The mean number of the pulse of the new-born infant is 123 and a fraction.
2. The maximum number in the above experiments was 160, and the minimum 100.
3. The mean number of three, who were stated in the table as being in the horizontal posture (for I believe all were horizontal, although I cannot aver this for a fact), was 124.
4. The average number of the pulse of three male infants, taken from the above experiments, was 120.
5. The pulse of one twin female was 116.

My first conclusion is derived from facts, without any allusion to the posture, the state of motion or rest, or muscular exertion. Neither do I know of any author who has noticed these particulars as regards the infantile state, and as connected with the pulse. My third conclusion is derived from three experiments: a scanty number, truly; yet more, indeed, than has been made by any one else, according to my reading.

II. *From one Day to one Week Old.* (42 Observations.)

1. The mean pulse of the infant between the age of one day and one week is 128.
2. The maximum in the above experiments is 160, the minimum 96.
3. The pulse of one female in the horizontal posture (although most of them were probably in the horizontal posture, yet it is not stated in the table, and must not, therefore, be given as a fact) was 120.
4. The mean number taken from eleven females is 131.71.
5. The mean number taken from nineteen males is 122. From four and five it results that the pulse of females is much more frequent than that of males.
6. The average of three experiments on children asleep, and in the horizontal posture, is 108.
7. The pulse is quicker during the first week than during the first day.
8. The pulse is slower during the first week than it is found to be afterwards.

III. *From one Week to one Month Old.* (31 Observations.)

1. The mean number of the pulse in the infant, from one week to one month old, is 135.45.
2. The maximum in the above table is 176, and the minimum 104.
3. The mean, from experiments on eight in the horizontal position, is 125: the mean of two of them which were asleep being 122, and of two which were twins, and not asleep, 108.
4. The mean, from experiments on six females, is 141.67.
5. The mean, from experiments on five females, is 130. From five and six it results that the pulse is quicker in females than in males.

From the second and third tables, which together include experiments on 73 infants, it results—that the pulse in the infant from one day to one month old, is 131.

IV. *From One to Five Months old.* (15 Observations.)

1. The mean pulse of the infant from one to five months old is 148.85.
2. The maximum number in the above experiments is 176, and the minimum 104.
3. The mean of five, which were in the horizontal posture, is 144; and of these, four were asleep, the mean number of the pulse being 140.
4. The mean of five, in the semi-recumbent position, is 151.20; and of these, three were sucking.
5. The pulse is much more frequent after the first month than before it; and this frequency increases up to a certain period.

V. *From five Months to two Years old.* (19 Observations.)

1. The mean pulse of the child, from the fifth month to the second year, is 130.
2. The maximum from these experiments is 158, and the minimum 100.
3. The mean of five experiments, in which the children were in the horizontal position and fast asleep, is 126.40.
4. The mean of five experiments, in which the children were sitting, is 142.
5. The mean of three experiments, in which the children were standing, is 128.33.
6. The pulse at this age, which embraces the teething period, is quicker than at birth.

VI. *From two to four Years old.* (14 Observations.)

1. The mean pulse in the child from two to four years old, is 112.56.
2. The maximum number in the experiments is 124, and the minimum 92.
3. The mean of four, who were in the horizontal position and asleep, is 102.
4. The mean of four who were standing, is 118.
5. The number of one who was sitting, is 120.
6. The pulse at this age, which has passed the teething period, is much slower; and from this time it gradually diminishes in frequency to the end of life.

VII. *From four to ten Years old.* (13 Observations.)

1. The mean pulse in the child between the fourth and tenth year, is 107.63.
2. The maximum in the above table is 133, and the minimum 88.
3. The mean of five experiments on those in the horizontal position, is 104.
4. The mean of six experiments on those who were standing, is 110.17.
5. The mean of four experiments on those who were sitting, is 107.50.
6. The pulse is slow in the horizontal posture. It acquires frequency if this be changed to a sitting, and becomes still more frequent in the erect position.

Mr. Gorham very candidly admits "that, although all the observations that have been hitherto made on the pulse of the infants are not without their value, yet that they are rendered imperfect and susceptible of improvement, by taking into consideration the circumstances of posture, and laws emanating therefrom, mentioned in the extremely valuable paper of Dr. Guy."

Owing to the inability of very young infants to preserve themselves in the sitting or erect position by means of the action of their own muscles, Mr. Gorham thinks that the acceleration of pulse witnessed on placing infants in the erect position must be attributed to some other cause than that adduced by Dr. Guy, viz. muscular contraction. We do not, however, coincide in this opinion; because infants, although incapable of *supporting themselves* by muscular contraction, may still employ this, and no doubt do so, to a greater extent than when laid in the horizontal position. *Med. Gazette. November* 25, 1837.

